# Stakeholder views on work participation for workers with depression and intersectoral collaboration in depression care: a focus group study with a salutogenic perspective

**DOI:** 10.1080/02813432.2023.2238019

**Published:** 2023-08-01

**Authors:** Heidi Marie Meling, Norman Anderssen, Sabine Ruths, Stefán Hjörleifsson, Inger Haukenes

**Affiliations:** a Research Unit for General Practice, NORCE – Norwegian Research Centre, Bergen, Norway; b Department of Global Public Health and Primary Care, University of Bergen, Bergen, Norway; c Department of Psychosocial Science, University of Bergen, Bergen, Norway

**Keywords:** Intersectoral collaboration, depression, sick leave, return-to-work, sense of coherence, focus groups, work participation

## Abstract

**Objective:**

To explore how stakeholders in depression care view intersectoral collaboration and work participation for workers with depression.

**Design:**

Focus group study applying reflexive thematic analysis using a salutogenic perspective.

**Setting and subjects:**

We conducted seven focus group interviews in six different regions in Norway with 39 participants (28 women); three groups consisted of general practitioners (GPs), two of psychologists and psychiatrists and two of social welfare workers and employers (of which one group also included GPs).

**Results:**

Stakeholders considered work participation salutary for most workers with depression, given the right conditions (e.g. manageable work accommodations and accepting and inclusive workplaces). They also highlighted work as an integral source of meaningfulness to many workers with depression. Early collaborative efforts and encouraging sick-listed workers to stay connected to the workplace were considered important to avoid long and passive sickness absences. Furthermore, stakeholders’ views illuminated why intersectoral collaboration matters in depression care; individual stakeholders have limited information about a worker’s situation, but through collaboration and shared insight, especially in in-person collaborative meetings, they (and the worker) can gain a shared understanding of the situation, thereby enabling more optimal support. Ensuring adequate information flow for optimal and timely follow-up of workers was also emphasized.

**Conclusions:**

Stakeholders highlighted the salutary properties of work participation for workers with depression under the right conditions. Intersectoral collaboration could support these conditions by sharing insight and knowledge, building a shared understanding of the worker’s situation, assuring proper information flow, and ensuring early and timely follow-up of the worker.

## Introduction

Common mental disorders like depression are a leading cause of years lived with disability worldwide [1]. Depressive disorders are highly prevalent with high recurrence rates [2] and are associated with the risk of long-term sick leave, work disability and labor market marginalization [3–5]. Knowledge contributing to facilitating work participation and a sustainable working life for workers with depressive disorders – be they mild or severe – is, therefore, highly warranted [6]. Insight into stakeholders’ views on depression care and return to work (RTW) efforts for workers with depression is crucial, as they are instrumental for these processes to be successful [7].

Most people with depression are treated in primary healthcare settings, but many determinants of health, such as work and social welfare, lie outside the purview of the healthcare sector. Intersectoral collaboration, here understood as cooperative action among stakeholders in healthcare, working life and social welfare services, has been shown to be effective in minimizing sickness absence, facilitating work participation, and aiding the RTW process for sick-listed workers with depression [8]. Intersectoral collaboration can also present challenges such as silo thinking, diverging interests, unsettled power balances and insufficient information flow between and within sectors [9–11]. Moreover, poor collaboration between healthcare professionals and employers has been shown to hinder work participation for workers with depression and may carry negative consequences for the worker [12].

In Norway, stakeholders commonly involved in intersectoral collaboration when a worker is sick-listed with depression are general practitioners (GPs) and/or psychologists or psychiatrists in secondary mental healthcare, employers (i.e. workplace managers) and social service workers. Most people with depression are treated by their GP [13], and about 99% of the Norwegian population has a designated GP [14]. If deemed necessary, GPs may refer their patients to treatment in secondary mental healthcare (e.g. due to insufficient effect of treatment in primary healthcare, detected suicide risk, etc.) [8]. Employers are involved because they are legally bound to make workplace accommodations for workers with reduced work ability and to follow up with sick-listed workers [15]. Furthermore, social welfare services are responsible for intermittent oversight of the RTW process, providing support for work accommodation, and issuing sick pay after the initial 16 days of sickness absence (which are covered by the employer) [16] (see Table 1 for an overview of follow-up of sick-listed workers in Norway).

**Table 1.** Follow-up of sick leave in Norway.

Sickness benefits: 100% of income covered for up to 12 months. Days 1–16 (covered by the employer) Day 17 up to 12 months (covered by the state)		
Stakeholder responsibilities during sick leave (maximum of 12 months)		
*General practitioner (GP)* Day 1 up to 12 months: Assess work capacity, need for sick leave and issuing sick-leave certification (graded or full). Can make non-disclosive suggestions to the employer for follow-up and work modifications. Weeks 8, 17, 26 and 39: Report patient status to social welfare services.	*Employer* Week 4: Establish a plan for work modifications and return to work (RTW) with the sick-listed worker. May share the plan with GP and social welfare services. Week 7: Arrange Dialogue meeting 1 with the worker to discuss status, RTW and update plan. Social welfare services and GP may be invited.	*Social welfare services* Day 1 up to 12 months: Issue sickness benefits. Week 26: Arrange Dialogue meeting 2 – a triparty meeting between the sick-listed worker, employer and social welfare services to assess the situation and make further plans for RTW. Any party may request this meeting to be held at an earlier time. If necessary, GPs are asked to attend but are often exempt.

Sickness benefits (income coverage) and stakeholder responsibilities during a worker’s sick leave.

Depression symptom reduction and RTW processes do not necessarily run parallel; for many, work participation can be an integral part of depression recovery [17]. Therefore, Norwegian GPs are urged to limit sick leave certification, especially in milder cases where work may be beneficial by providing meaningful everyday activity and social interaction [8]. Drawing upon the salutary (i.e. health-promoting) effects of work echoes salutogenic theory, which aims to understand how people can move toward health and well-being wherever they find themselves on the health/disease continuum [18]; thus, salutogenesis may provide a valuable perspective to explore issues of sick leave and RTW for workers with depression. In salutogenic theory, work is considered a potentially salutary determinant of health as long as it supports a person’s sense of coherence (SOC) [19]. SOC refers to a person’s perception of life as comprehensible, manageable and meaningful [18,20]. A stronger SOC enables people to identify and apply their internal and external resources to meet life challenges and improve and/or maintain good health [21]. People with stronger SOC have been shown to have better health than those with weaker SOC [22]. As work is considered an important life setting for shaping a person’s SOC, and by extension, their health [19,20], a work-specific SOC, work-SOC, has been proposed by Bauer and Jenny [23], referring to an individual’s perception of comprehensibility, manageability and meaningfulness in their work situation. Associations have been found between work-SOC and general SOC, and between resourceful working conditions and higher work-SOC [24,25].

Several studies have investigated RTW interventions for workers with common mental disorders and collaboration among involved stakeholders [26–28], whose views and experiences are essential to understanding and promoting successful RTW interventions. However, as we reviewed the RTW literature, we observed a lack of research investigating the views of depression care stakeholders in a Scandinavian setting that included the social welfare services in the stakeholder matrix. Also, to our knowledge, no studies have explored these stakeholders’ views using a salutogenic perspective, which might offer valuable insights. Thus, this study aimed to use a salutogenic perspective to explore the views and experiences of stakeholders in depression care in Norway regarding work participation for workers with depression and how intersectoral collaboration may support them. Our research question was twofold: (1) what are Norwegian depression care stakeholders’ views on work participation for workers with depression? and (2) what are their views on how intersectoral collaboration can support work participation for workers with depression?

## Materials and methods

The multidisciplinary research team had extensive and complementary experience in social science (H.M.M.), general practice (S.H. and S.R.), psychology (N.A.) and physiotherapy (I.H.). We chose focus group interviews as a method of data collection, as they are useful when exploring shared experiences, attitudes and reflections in contexts where people interact [29]. Uncovering potential diverging opinions or consensus may be informative, and the group setting, where participants can respond to and build upon each other’s contributions, can create synergetic effects that can generate data individual interviews may not capture [30].

### Setting and sample

We employed a snowball method to recruit a purposive convenience sample starting with our extended professional networks. Participants were invited to the study via telephone and email by H.M.M. with assistance from I.H. and S.H. The sample consisted of 39 participants (28 women, 11 men): 22 GPs, three psychologists, three psychiatrists, four social welfare workers and seven employer representatives (i.e. managers) from local workplaces. We allocated participants to seven focus group interviews conducted in six geographically spread regions of Norway (urban and rural areas). For participant and group characteristics, see Table 2.

**Table 2.** Focus group characteristics.

Group number	Participants	Geographical setting
1	2 psychologists − 1 woman, 1 man 1 psychiatrist – man	Urban
2	1 Psychologist – woman 2 Psychiatrists – women	Urban
3	8 GPs^a^ − 6 women, 2 men	Rural
4	7 GPs − 3 women, 4 men	Rural
5	4 GPs − 3 women, 1 man	Urban
6	3 GPs − 1 woman, 2 men 2 social welfare workers – women 4 employer representatives – women	Rural
7	2 social welfare workers – women 3 employer representatives – women	Rural
*Total number of participants:* 39 (28 women, 11 men)		

### Data collection

We conducted all focus group interviews between January and March 2020 in local settings convenient to the participants, such as the GPs’ offices, local social welfare services offices, etc. The interviews lasted between 50 and 90 min. Four research team members, H.M.M., N.A., S.H. and I.H., conducted the focus groups in pairs, with one team member moderating and the other co-moderating. In six of the seven groups, H.M.M. functioned as moderator. S.H. moderated the remaining group. The co-moderators took notes and supplied the moderator with follow-up questions if needed.

We piloted our interview guide with a group of three GPs who were not a part of the study sample. The interview guide consisted of topics regarding work participation for workers with depression and intersectoral collaboration in depression care. Throughout the data collection, we adjusted the interview guide to follow up on topics raised in concluded focus groups and accommodated issues relevant to the stakeholders in each focus group. Examples of topic questions from the interview guide were ‘What are your reflections regarding work participation for workers with depression?’ or ‘How would you describe your experiences with intersectoral collaboration in depression care?’. We conducted the focus groups as open discussions among participants, where they shared views, experiences and reflections on the given topics. We opened the first four focus groups by using a hypothetical case vignette. The vignette described a 48-year-old female nurse on 50% sick leave due to depression for the past five months. Her current depressive episode started after her father passed away, and she experienced workplace conflicts at the care facility where she worked. Her GP was familiar with her history of depression and consulted with her once a month. We later discarded the vignette to home in on participants’ own experiences and reflections. All focus group interviews were audio-recorded. Each completed focus group interview was reviewed before succeeding focus groups were held.

### Data analysis

Audio recordings of the focus group interviews were transcribed verbatim and anonymized by H.M.M. Transcription and data analysis were performed using the qualitative data analysis software NVivo. Reflexive thematic analysis was applied to establish shared patterns of meaning within and across the data [31,32]. H.M.M., N.A. and I.H. familiarized themselves with the data through repeated readings of the transcripts. Subsequently, a hybrid of data-driven (inductive) and theory-driven coding was used [33], where H.M.M. first performed inductive, open, comprehensive coding of the transcripts in consultation with N.A. and I.H. identifying every instance of these codes across the data set. Based on this first data-driven round of coding, H.M.M. and I.H. discussed candidate themes, after which H.M.M. re-engaged with the data and performed more targeted theory-driven coding, considering how the data material could be understood in a salutogenic perspective, especially how the coding from the first round could be illuminated by the three elements of SOC, meaningfulness, manageability and comprehensibility. H.M.M. and I.H. further discussed and developed candidate themes before refining and defining the themes presented in the results. S.H. and S.R. were consulted during the development and refinement of themes. An example of the coding process can be found in Appendix 1.

## Results

We generated two themes through the analysis. The first theme, ‘The salutary value of work, given the right conditions’, described the participant’s general views of work participation as health-promoting, some of their caveats regarding necessary conditions to consider, and views on how intersectoral collaboration can play a role. The second theme, ‘How intersectoral collaboration can help support workers with depression’, further illustrated why and how intersectoral collaboration matters in relation to depression care, work participation and RTW for workers with depression.

### The salutary value of work, given the right conditions

Overall, participants viewed work participation as health-promoting for most workers with depression, given the right conditions, the worker’s symptom severity and level of functioning. Several healthcare professionals had experienced that, for some patients, work participation was not manageable at the onset of treatment due to symptom severity and low level of functioning. Still, they emphasized the need to consider RTW as an integral part of the depression recovery process from the start, at least as a goal, and raise the matter as soon as they deem it appropriate for the patient. Participants noted the necessity of encouraging sick-listed workers to maintain a connection with the workplace (e.g. working reduced hours, joining workplace lunches or other social interactions with colleagues) and help workers with depression feel included and valued in the workplace, regardless of their health status. However, participants also noted that depression might be exacerbated or triggered in the workplace:

I think it’s a common understanding that work is health-promoting for most, but sometimes part of the issue [of depression] manifests in the workplace. If that’s the case, it may not be as easy to return, and maybe a meeting with the employer would be appropriate. (Rita, Psychologist, FG 2)

In all the focus groups, participants discussed how workplace conflicts, poor personnel management and toxic work relationships (especially between workers and employers) could hinder work participation and/or RTW and complicate recovery. A few psychologists/psychiatrists experienced that they had to help some patients consider changing workplaces or uncover and mend unhealthy work relationships to improve the interaction between their work-life and mental health. Participants also shared examples of cases where they had perceived employers as highly proactive, solution-oriented and flexible in their accommodation of workers with depression, showing a strong focus on supporting the worker’s sense of manageability:

I had a case where a man had been severely depressed over time. He couldn’t do his job or answer enough calls or emails. It all piled up, exacerbating his depression. However, his boss had taken a course on sick leave management. So, when the man returned to work, his boss had fixed everything! He let the man choose his work responsibilities and tasks and filtered his calls and emails so that he would only have to deal with issues related to these chosen tasks as he started back up. (Liv, Psychologist, FG 1)

Participants also noted the need to promote acceptance of natural fluctuations in work performance for workers with depression. Some healthcare professionals discussed experiences where workers had been reluctant to RTW if they did not feel confident their work performance would be optimal. One social welfare worker explained that a vital part of their follow-up of sick-listed workers was to help establish their ‘true’ work capacity:

If you’re repeatedly sick-listed, maybe we need to figure out what your capacity to work really is, and perhaps it is lower; you return to work, manage for a while, and then fall back out again. Maybe, in the long run, you would function better if you had reduced your work a bit over time. (Irene, Social welfare worker, FG 7)

The social worker’s statement suggests a salutogenic approach by finding ways in which people can improve or maintain their health despite – but not regardless of – their health issues. One can also see how working to establish and accommodate a worker’s ‘true’ work capacity can support manageability, comprehensibility and meaningfulness (i.e. SOC); if work is made to be more manageable over time, work participation might make more cognitive sense, and thereby feel like a more meaningful endeavor.

### How intersectoral collaboration can help support workers with depression

As the participants indicated, making work participation salutary for workers with depression may require the involvement and collaboration of several stakeholders for several reasons. For one, workers may feel comfortable discussing depression with a healthcare professional but be reluctant to disclose depression as the reason for their reduced work capacity or sick leave to their employers due to stigma and taboos surrounding mental illness. One employer stated in reference to privacy matters: ‘Totally different things may be communicated to us, and that is completely natural!’ (Ruth, Employer, FG 7). Still, employers tended to experience it as more challenging to support workers who were not open about their illnesses and needs. Healthcare professionals were also sensitive to issues of stigma when they discussed with their patients whether open communication and disclosure in the workplace could be beneficial: ‘Depending on what your job is, it might not always be a good idea to expose issues like this at work’ (Helen, GP, FG 5). Several participants highlighted the need for employers to be proactive and take preventive measures to create a workplace where open communication felt natural and safe. This could make both workers and employers better prepared for situations where workers might experience reduced work capacity due to depression, need work modifications or general support in a RTW process:

It might be more difficult for a worker to talk about depression than a broken arm. But I believe it’s about your connection with the worker *before* sick leave because you might pick up on signals that things are not okay. So, I think we ought to work together, during sick leave, after sick leave, always really, to be able to pick up on symptoms. A sick listing should not come as a surprise. (Rose, Employer, FG 6)

Second, some participants were concerned that many employers do not possess the knowledge, skills or capacity to support workers with depression and enable work participation optimally. The employer’s role in the RTW process was emphasized in all focus groups. Some GPs shared positive experiences with engaging in the worker/employer dialogue:

If we get the employer involved, it works out well almost every time. If I can, in some way, get the employer to understand me and my patient’s position. Of course, that’s contingent on the employer’s willingness to get invested. (Donna, GP, FG 5)

GPs have information about their patient’s health and life circumstances, while their patient’s employers have information about their work life, but neither necessarily has much insight into the information held by the other (and, notably, privacy issues must be carefully navigated). Still, both hold crucial information that carries more potential for optimal care when used together in a depression recovery and RTW process. Several employers remarked on how healthcare professionals and other stakeholders could support them in promoting work participation and RTW. For instance, one employer shared positive experiences contacting social welfare services for advice on managing sick-listed workers. This participant also brought up the challenge of determining how much to expect or demand of a depressed and/or sick-listed worker due to a lack of insight into her workers’ health issues. Some GPs said they used the sick-leave certification to make suggestions for work modifications and that they sometimes used them with the specific aim of managing the employer’s expectations, for example, by writing: ‘At the moment, the patient is having a lot of problems and cannot be expected to do very much’ (Robert, GP, FG 3). Though many employers were favorable to this practice, one of them sometimes felt overrun by GPs telling them how to accommodate their workers in unrealistic ways, pointing to GPs’ lack of information about their patients’ work life. The way some employers described efforts to accommodate workers with depression implied a balancing act between wanting to help and meeting workplace demands:

Regarding returning to work, which tasks the worker can perform, and work modifications, we can’t always accommodate the worker’s wishes, as we have certain workplace demands. (Rose, Employer, FG 6)

Challenges in depression care were frequently discussed by the participants and often attributed to fragmented healthcare- and welfare systems and poor information flow between stakeholders (even within sectors). The perception was that many of these challenges could have been alleviated or avoided through tighter and more timely collaboration. For example, healthcare professionals described the necessity of being up to date on their patient’s status to ensure progression in treatment and the RTW process if they were on sick leave. However, GPs in all groups had experienced receiving little to no updates on their patients’ status while treated in secondary mental healthcare. The GPs often received a case summary only *after* the patient had finished treatment, which could take months. Consequently, it could be difficult for the GPs to coordinate with other stakeholders, for example, when social welfare services requested documentation of the health status of patients being evaluated for social welfare schemes (e.g. work assessment allowance or disability pension):

I had a situation today regarding a long-term sick leave where I asked for a report of my patient’s stay [in secondary mental healthcare], but I haven’t received it. Now I’m supposed to provide documentation of the patient’s health status [to social welfare services]. The patient has also seen an occupational psychologist from social welfare services, who hasn’t sent me anything. It’s very strange. It’s almost laughable. Are we supposed to be guessing here? (Donna, GP, FG 5)

Such experiences exemplify how poor information flow can impede individual stakeholders’ ability to follow up with their patients in a timely manner. Meanwhile, as one of the social welfare workers pointed out, the worker’s sickness absence may be unnecessarily prolonged. One might see how delayed follow-up can create uncertainty for the worker and, in light of SOC, negatively affect their sense of comprehensibility and manageability in their RTW process.

Examples of how intersectoral collaborative efforts could positively support a worker’s sense of comprehensibility and manageability also came to light. For instance, several participants found in-person collaborative meetings helpful in building a shared understanding of a worker’s situation and allocating responsibilities, finding solutions to work modifications, and making plans for RTW:

The meetings formalize collaboration between social welfare services, the employer, the patient, and the GP. Sometimes, I experience more seriousness in placing responsibilities, especially with employers. Also, everything is put in writing, so such meetings can help push things in a positive direction. (Edward, GP, FG 6)

Many participants stressed the need to encourage and support work participation earlier in the stages of sick leave, as lengthy and passive sick leave was perceived to be detrimental to recovery and RTW. One social welfare worker pointed out how collaboration between GPs and the workplace could be central to avoiding passive sick leave:

After all, GPs are the ones issuing the sick-leave certificates. You’ve [GPs] got to make evaluations as you write them up and make suggestions to employers and social welfare services if something should be done; if not, it may result in a very passive sick leave that keeps going. (Carol, Social welfare worker, FG 6)

Some used the risk of prolonged and passive sick leave as an argument for collaborative meetings to be held earlier. One employer highlighted how early collaboration between themselves, and GPs could promote work participation and possibly avoid unnecessary sick listings. They also wished for GPs to initiate dialogue even before sick listing their patients to explore possibilities for work modifications:

Sometimes sick-leave certifications tumble in without us even getting a chance to say: ‘Here we could have made work modifications and avoided sick-leave.’ (Hanna, Employer, FG 7)

In sum, the participants illuminated how individual stakeholders, none of whom have complete information about a worker’s situation, can provide better support for workers with depression through sharing knowledge, skills and information in intersectoral collaboration.

## Discussion

### Main findings

In this focus group study, we posed the following research questions: (1) what are Norwegian depression care stakeholders’ views on work participation for workers with depression? and (2) what are their views on how intersectoral collaboration can support work participation for workers with depression? We further explored how these stakeholder views could be understood from a salutogenic perspective using the three elements of SOC, meaningfulness, manageability and comprehensibility. Stakeholders viewed work participation as salutary under the right conditions. According to the participants, intersectoral collaboration could support these conditions by sharing insight and knowledge, building a shared understanding of the worker’s situation, assuring proper information flow, and ensuring early and timely follow-up of the worker.

### Findings in light of salutogenic theory and existing research

Most research on collaboration in depression care has focused on intrasectoral collaboration between healthcare professionals, whereas our study broadens the scope to intersectoral collaboration. A review of the effectiveness of workplace intervention in RTW showed strong evidence that multi-faceted interventions operating across multiple domains (health focus, service coordination and work modification) effectively improved outcomes in workers with mental health conditions [34]. Our findings relating to how gathering information through intersectoral collaboration holds a higher potential for optimal depression care align with the findings of that review, as well as the call for the inclusion of social welfare services and workplaces when investigating and providing depression care. This wider scope also corresponds with the salutogenic approach of seeing individuals embedded in the context of their social world, the resources that are (un)available to them, and how it affects their ability to improve their health [18,20]. Participants in this study noted how intersectoral collaborative efforts in depression care could benefit patient outcomes, work participation and RTW, which is in line with previous research [27]. Our participants described how having in-person intersectoral collaborative meetings could support comprehensibility and manageability for the worker. This finding may relate to the two central mechanisms underlying SOC, suggested by Super et al. [35]: a perceptual mechanism, where reflection is essential to promote an understanding of the stressors people face and the resources they have available to cope with them, and a behavioral mechanism, which highlights possibilities to empower people to take advantage of their resources to cope with stressors. As our participants described, collaborative meetings can help build a shared understanding of the worker’s situation and available resources, enabling reflection and thereby engaging the perceptual mechanism. In such meetings, the allocation of resources and responsibilities can be formalized between stakeholders in the RTW process to benefit the worker, empowering them and thereby engaging the behavioral mechanism.

According to Antonovsky: ‘[…] the strength of the sense of coherence […] can be modified, detrimentally or beneficially, by the nature of the current working environment‘ [19]. Though participants in the present study considered work participation salutary for most workers with depression, they presented the caveat ‘given the right conditions.’ As the participants further emphasized, employers can be instrumental in intersectoral collaboration to create the conditions needed for work participation to be salutary. The employers’ role in working for workplaces to be accepting, inclusive and accommodating for depressed workers underscores the importance of including employers as stakeholders when investigating depression care and the RTW processes, which is in line with RTW research underlining the workplace component [12,34,36].

The study participants stressed the benefit of employers focusing on preventive measures, such as fostering supportive workplace relationships, building constructive and open workplace dialogue, and an inclusive work environment where workers feel appreciated, regardless of their health status. The benefits of building such psychosocial job resources are supported in the RTW literature [37] and may help create an environment where depressed workers’ sense of comprehensibility, manageability and meaningfulness can be strengthened. Stigma related to mental illness is a known detriment to psychosocial work conditions [12], a concern raised by stakeholders in the present study. A study among people with depression by Brouwers et al. [38] supports their concern and their emphasis on the need for inclusive and accommodating workplaces; 62.5% of participants (*N* = 834) reported experiences of, or anticipated experiences of, discrimination in the work setting. The findings led researchers to conclude that interventions to enhance work participation in people with depression must focus on decreasing stigma in the work environment and self-discrimination due to anticipated discrimination.

As noted by Jenny et al., work constitutes a significant part of life for most, making working conditions important determinants of SOC and, by extension, a person’s, a family’s – even a community’s health [39]. Though work may be health-promoting, a notion shared by the participants in the present study, it is crucial to consider the intersection between individuals’ work and personal life, especially in relation to depression care. In a Swedish phenomenological study on the capacity to work while depressed and anxious [40], researchers found that participants experienced difficulties ‘recharging’ through positive activities and social interactions in their spare time because they spent so much energy maintaining their capacity to work. These findings suggest that work capacity, and by extension, work participation, cannot be viewed in a vacuum; other aspects of life should be considered; for example, work-related stressors, such as balancing work and home demands, could have negative health effects. Our findings indicate that intersectoral collaboration can enable stakeholders in depression care to make more holistic considerations regarding a worker’s situation in their efforts to support them through the power of shared information.

### Strengths and limitations of the study

The multidisciplinary background of the research team constitutes a strength of this study, as it brought different perspectives to the topics at hand. We evaluated the size and composition of our final sample in data collection to have adequate information power [41] because the specificity of our sample was dense (i.e. the participants had a high capacity to elucidate the research aim); we applied salutogenic theory in analysis; the quality of dialogue in the focus groups was strong with participants openly sharing views, reflections and experiences; participants differed in age, experience and geographical setting, sufficiently to provide enough sample variation to provide both breadth and depth of perspectives.

Reflexivity was maintained by continuously re-engaging with the data throughout the research process. Also, H.M. regularly wrote reflection notes to uncover theoretical assumptions and preconceptions of the material and frequently discussed these with the other authors.

The topic of this study was professional stakeholders’ views; thus, we did not include patient voices. Other studies in the research project, The Norwegian GP-DEP Study, of which the current study is a part, do, however, investigate patient perspectives.

### Implications for further research and practice

The findings in this study point to a need for intervention studies examining the effect of intersectoral collaboration in depression care, focusing on creating salutary conditions for work participation and RTW for workers with depression. While this study broadened the collaborative matrix of depression care to include stakeholders in social welfare services and workplaces, future studies should include a wider array of stakeholders with a clear workplace component, e.g. professionals in occupational health services and workplace colleagues. The findings also have implications for policy and practice; they highlight the need for the Norwegian healthcare system to provide arenas, platforms, procedures and incentives for early and timely intersectoral collaboration in depression care and the need for employers to be included in such collaborative efforts, to better aid the RTW-process for workers sick-listed due to depression.

## Appendix 1. Example of coding

**Table:**

Data excerpt	Code(s)	Theme
So, when the man returned to work, his boss had fixed everything! He let the man choose his work responsibilities and tasks and filtered his calls and emails so that he would only have to deal with issues related to these chosen tasks as he started back up.	Work accommodation Employer follow-up Manageability Workplace conditions conducive to work participation	The salutary value of work participation, given the right conditions
